# Emergence of a Peaceful Culture in Wild Baboons

**DOI:** 10.1371/journal.pbio.0020124

**Published:** 2004-04-13

For most animal species, behavioral attributes are largely the product of interactions between genes and environment, with behavioral patterns preserved by natural selection. Birds, for example, know instinctively what type of nest to build for their offspring; salamanders don't need lessons to swim. But when it comes to primates—including humans—a good deal of behavior is learned. Primates exhibit a wide range of behaviors, not just among species but also among populations and even individuals. Yet the nature versus nurture debate still rages, particularly when it comes to understanding the roots of aggression. While bonobos are famous for using sex to resolve disputes, aggression is far more common in most primate species—again humans included. Our closest relative, the chimpanzee, has a reputation for being among the most belligerent, with rhesus monkeys and baboons not far behind. For many of these species, bouts of violence are often followed by gestures of reconciliation, such as grooming or, in the case of chimps, kissing. Since most primates live in social groups, it may be that such conciliatory measures serve to maintain some semblance of social structure, offsetting the disruptive effects of aggression. (To learn more about primate behavior and aggression, see the primer by Frans de Waal in this issue [DOI: 10.1371/journal.pbio.0020101].)[Fig pbio-0020124-g001]

**Figure pbio-0020124-g001:**
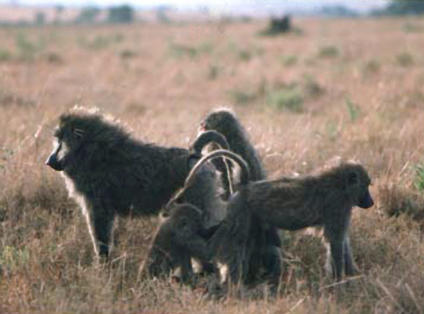
In baboons, “grooming” is a socially rewarding behavior. (Photograph, with permission, by Robert Sapolsky)

Primatologists characterize these behavioral differences as “cultural” traits, since they arise independent of genetic or environmental factors and are not only shared by a population (though not necessarily a species) but are also passed on to succeeding generations. Such cultural traditions have been documented in African chimp populations, which display over 39 behaviors related to “technology” (such as using stones to crack nuts), grooming, and courtship. While most of these cases involve either tools, foraging, or communication, Robert Sapolsky and Lisa Share report evidence of a higher order cultural tradition in wild baboons in Kenya. Rooted in field observations of a group of olive baboons (called the Forest Troop) since 1978, Sapolsky and Share document the emergence of a unique culture affecting the “overall structure and social atmosphere” of the troop.

In his book *A Primate's Memoir*, Sapolsky studied the activities and lifestyle of the Forest Troop to explore the relationship between stress and disease. In typical baboon fashion, the males behaved badly, angling either to assume or maintain dominance with higher ranking males or engaging in bloody battles with lower ranking males, which often tried to overthrow the top baboon by striking tentative alliances with fellow underlings. Females were often harassed and attacked. Internecine feuds were routine. Through a heartbreaking twist of fate, the most aggressive males in the Forest Troop were wiped out. The males, which had taken to foraging in an open garbage pit adjacent to a tourist lodge, had contracted bovine tuberculosis, and most died between 1983 and 1986. Their deaths drastically changed the gender composition of the troop, more than doubling the ratio of females to males, and by 1986 troop behavior had changed considerably as well; males were significantly less aggressive.

After the deaths, Sapolsky stopped observing the Forest Troop until 1993. Surprisingly, even though no adult males from the 1983–1986 period remained in the Forest Troop in 1993 (males migrate after puberty), the new males exhibited the less aggressive behavior of their predecessors. Around this time, Sapolsky and Share also began observing another troop, called the Talek Troop. The Talek Troop, along with the pre-TB Forest Troop, served as controls for comparing the behavior of the post-1993 Forest Troop. The authors found that while in some respects male to male dominance behaviors and patterns of aggression were similar in both the Forest and control troops, there were differences that significantly reduced stress for low ranking males, which were far better tolerated by dominant males than were their counterparts in the control troops. The males in the Forest Troop also displayed more grooming behavior, an activity that's decidedly less stressful than fighting. Analyzing blood samples from the different troops, Sapolsky and Share found that the Forest Troop males lacked the distinctive physiological markers of stress, such as elevated levels of stress-induced hormones, seen in the control troops.

In light of these observations, the authors investigated various models that might explain how the Forest Troop preserved this (relatively) peaceful lifestyle, complete with underlying physiological changes. One model suggests that nonhuman primates acquire cultural traits through observation. Young chimps may learn how to crack nuts with stones by watching their elders, for example. In this case, the young baboon transplants might learn that it pays to be nice by watching the interactions of older males in their new troop. Or it could be that proximity to such behavior increases the likelihood that the new males will adopt the behavior. Yet another explanation could be that males in troops with such a high proportion of females become less aggressive because they don't need to fight as much for female attention and are perhaps rewarded for good behavior. But it could be that the females had a more direct impact: new male transfers in the Forest Troop were far better received by resident females than new males in the other troops.

Sapolsky and Share conclude that the method of transmission is likely either one or a combination of these models, though teasing out the mechanisms for such complex behaviors will require future study. But if aggressive behavior in baboons does have a cultural rather than a biological foundation, perhaps there's hope for us as well.

